# Heroin use impairs smoking cessation among Australian prisoners

**DOI:** 10.1186/1471-2458-13-1200

**Published:** 2013-12-19

**Authors:** Devon Indig, Alex D Wodak, Robyn L Richmond, Tony G Butler, Vicki A Archer, Kay A Wilhelm

**Affiliations:** 1 Justice & Forensic Mental Health Network, Centre for Health Research in Criminal Justice, Suite 302, Level 2, 152 Bunnerong Road, Pagewood, NSW 2035, Australia; 2 School of Public Health and Community Medicine, University of New South Wales, Sydney, NSW 2052, Australia; 3 Alcohol and Drug Service, St Vincent’s Hospital, 390 Victoria Street, Sydney, NSW 2010, Australia; 4 Kirby Institute, University of New South Wales, Sydney, NSW 2052, Australia; 5 Faces in the Street, Institute of Urban Mental Health and Wellbeing, St Vincent’s Hospital, Level 6, O’Brien Centre, 390 Victoria Street, Sydney, NSW 2010, Australia; 6 School of Psychiatry, University of New South Wales, Sydney, NSW 2052, Australia

**Keywords:** Heroin, Prisoner, Smoking cessation, Nicotine dependence

## Abstract

**Background:**

Prisoners have extremely high rates of smoking with rates 3–4 times higher than the general community. Many prisoners have used heroin. The aims of this study were to investigate the impact of heroin use on smoking cessation and the social determinants of health among prisoners.

**Methods:**

Secondary analysis of data from a randomised controlled trial of a multi-component smoking cessation intervention involving 425 Australian male prisoners. Inmates who, prior to imprisonment, used heroin regularly were compared to those who did not use heroin regularly. Self-reported smoking status was validated at baseline and each follow-up by measuring carbon monoxide levels. Readings exceeding 10 ppm were defined as indicating current smoking.

**Results:**

Over half (56.5%) of the participants had ever used heroin while 37.7% regularly (daily or almost daily) used heroin in the year prior to entering prison. Prisoners who regularly used heroin had significantly worse social determinants of health and smoking behaviours, including lower educational attainment, more frequent incarceration and earlier initiation into smoking. Prisoners who regularly used heroin also used and injected other drugs significantly more frequently. At 12-month follow-up, the smoking cessation of prisoners who had regularly used heroin was also significantly lower than prisoners who did not regularly use heroin, a finding confirmed by logistic regression.

**Conclusions:**

Regular heroin use prior to imprisonment is an important risk factor for unsuccessful attempts to quit smoking among prisoners and is also associated with worse social determinants of health, higher drug use, and worse smoking behaviours. More effective and earlier smoking cessation interventions are required for particularly disadvantaged groups.

**Trial registration:**

This trial is registered with the Australian New Zealand Clinical Trials Registry 12606000229572.

## Background

Smoking cessation efforts among the Australian community have led to a decline in smoking prevalence from a high of 72% of men and 26% of women in 1945 to a current regular smoking rate of 22% in men and 18% in women [1,2]. However, this trend is not seen in socially disadvantaged populations, such as prisoners, those with a mental illness, and illicit drug users [3-5]. The 2009 New South Wales (NSW) Inmate Health Survey, which randomly sampled 996 prisoners in custody, reported 75% of male and 80% of female prisoners were current smokers [6]. Among those entering prisons across Australia, the rate of smoking was higher (85%), and highest among prisoners reporting a history of injecting drug use (93%) [7]. However, a substantial proportion of prisoners report wanting to stop smoking [3,6,8,9].

The 2012 World Drug Report estimates that between 0.6% and 0.8% of the world’s population aged between 15 and 64 years used opioid drugs with heroin the most widely used opioid [10]. In Australia in 2010, among those aged 14 and over 1.4% reported using heroin while 0.2% used heroin in the previous year [1]. The rate of heroin use was significantly higher among prisoners in NSW with 41% reporting having ever used heroin, 10% using heroin on a daily/almost daily basis in the past year and 15% having ever used heroin in prison [6]. Among Australian prison entrants who participated in a National Prisoner Health Census in 2009, 19% of prisoners reported using heroin in the year before entering prison [11]. Heroin use among prisoners and the broader Australian community has decreased in the past decade following the heroin shortage of 2000, but rates are still high [12,13].

Most people who currently use heroin or are on opioid substitution treatment (such as methadone or buprenorphine) also smoke cigarettes [9]. A recent Australian study found the prevalence of smoking was 84% among methadone users [14] and another study of heroin users reported 97% were smokers [15]. A qualitative study [16] asked drug users currently in treatment about the relationship between their smoking, methadone and drug use. The participants reported that drug use and smoking were complementary in managing side effects of both substances and withdrawal symptoms [16]. Smoking cessation rates among opioid-dependent people are lower than among the general population, however many still want to quit [17,18].

Few studies have investigated how heroin use may affect smoking cessation intervention outcomes [19]. The aims of this paper were to assess the impact of regular heroin use in the year before entering prison on the social determinants of health, smoking behaviours (such as age of initiation and amount smoked per day) and success of quitting smoking among prisoners. As a high proportion of prisoners are current smokers and have used heroin, it is important that we improve our understanding of the relationship between the two in order to develop more effective smoking cessation interventions for this high risk and socially disadvantaged population.

## Methods

### Study design

This study involved a secondary analysis of data from a randomised controlled trial of a multi-component smoking cessation intervention among prisoners. This analysis compared prisoners who, prior to imprisonment, were regular (daily or almost daily use) heroin users with prisoners who were not regular heroin users (i.e. used heroin less than regularly). The trial recruited 425 male prisoners who received 13 weeks of nicotine replacement therapy, two sessions of brief cognitive behavioural therapy, had access to a Quitline, and were provided with smoking cessation support materials (e.g. a booklet designed by the investigators to assist with coping with prison stressors and a QUIT calendar). Participants were randomised to receive either active nortriptyline or placebo to assess the impact of the antidepressant on assisting participants to quit smoking. Ethics committees from the University of New South Wales, Justice Health NSW, Corrective Services NSW, the Aboriginal Health and Medical Research Council and Queensland Corrective Services approved the study. A full description of the methods, study intervention and results of the randomised controlled trial are reported elsewhere [20].

### Participants and recruitment

Recruitment took place in 17 prisons in NSW (N = 407) and one prison in Queensland (N = 18) among prisoners who were current smokers and who indicated they wished to quit smoking. Between August 2006 and September 2009, 1,751 prisoners expressed interest in participating in the study and were assessed by a research nurse and doctor regarding the selection criteria. The majority (N = 1,315) were considered ineligible for the following reasons: having less than six months left on their sentence (35%), being on psychiatric medication or having a history of psychiatric illness (40%) or a history of cardiac disease (5%). Female prisoners were considered ineligible, as they only constitute 7% of the NSW prisoner population and generally have shorter sentences [21]. All participants provided written informed consent and were paid $10 for each interview completed. Participants were followed up both in prison and the community at 3, 6 and 12 months to assess the point prevalence, continuous abstinence and smoking reduction. Follow-up rates were 90% at 3 months, 87% at 6 months, and 80% at 12 months, with no difference in follow-up rates for participants in the active nortriptyline and placebo groups. There was also no significant difference among participants who were regular or not regular heroin users regarding their assignment to treatment or placebo group, or with regard to their rate of follow-up.

### Questionnaires and measures

Participants were administered a baseline questionnaire face-to-face by research nurses. The baseline questionnaire included questions on socio-demographics, offending history, smoking behaviours, smoking history, physical health, mental health and alcohol and other drug use. The follow-up questionnaires included most of the same measures in the baseline. The questions related to use of heroin (and all drugs) including if they ‘ever used’ or ‘used regularly (daily/almost daily) in the 12 months before prison’. Smoking behaviours were measured using questions related to the number of cigarettes smoked before prison, years of regular smoking and prior quit attempts. Nicotine dependence was assessed using the Fagerström Test for Nicotine Dependence [22].

The primary smoking cessation outcome measures were point prevalence, continuous abstinence and 50% reduction in number of cigarettes smoked (relative to baseline) measured at 12 months. These outcome measures were determined on an intention-to-treat basis [23]: if a participant missed a particular follow-up they were regarded as a smoker at that time period. Self-reported smoking status was validated at baseline and each follow-up by measuring carbon monoxide levels and readings exceeding 10 ppm were classified as a smoker [24].

### Statistical analysis

Questionnaires were double-key entered into an electronic database and the data were cleaned for potential errors. Statistical analysis was performed using SAS version 9.2 [25]. Descriptive and inferential statistics (including chi-square and t-tests) were used to determine any statistically significant differences (p ≤ 0.05) across a range of characteristics for inmates who regularly used heroin compared with those who had not regularly used heroin in the year before prison. For the smoking cessation outcome variables, intention-to-treat analyses [23] were utilised with missing data classified as either continuing smoking or not reducing the amount smoked by 50%. This included chi-square analysis and logistic regression for the major outcome variables of point prevalence and continuous abstinence at 12 months. There were no significant differences in the follow-up rates for regular versus non-regular heroin users. In the logistic regression models, potential confounders were controlled for including any socio-demographics and other drug use characteristics which were significant.

## Results

### Socio-demographics, drug use and offending history by regular heroin users

Among the 425 male participants, over half (56.5%) had ever used heroin and 37.7% regularly (daily/almost daily) used heroin in the 12 months before entering prison. Prisoners who had regularly used heroin before prison were significantly younger than those who had not used heroin regularly (Table 1). Other socio-demographic characteristics which were significantly worse for prisoners who were regular heroin users included: institutionalisation as a child, leaving school with no qualification and at a younger age and being homeless prior to entering prison.

**Table 1:** Socio-demographics, drug use and offending history by regular use of heroin in year before prison

	**Not regularly used heroin % (N** = **265)**	**Regularly used heroin % (N** = **160)**	**Total men % (N** = **425)**	**P-value**
**Socio-demographics**				
Age group				
< 25 years	25.3	19.4	23.1	
25-29 years	17.4	24.4	20.0	P < 0.01
30-39 years	23.4	37.5	28.7	
40+ years	34.0	18.9	28.2	
Mean age	34.4	32.0	33.5	P < 0.02
(+SD; Range)	(11.2; 18–65)	(8.0; 19–54)	(10.2; 18–65)	
Aboriginal origin	14.3	16.3	15.1	NS
Born in Australia	74.8	75.0	74.9	NS
Left school no qualification	39.3	50.6	43.5	P < 0.03
Mean age left school	15.5	14.6	15.1	P < 0.01
(+SD; Range)	(1.7; 12–26)	(2.3; 0–19)	(2.0; 0–26)	
Institutionalised as a child (juvenile detention and/or placed in care)	31.3	48.8	37.9	P < 0.01
Homeless prior to prison	4.5	11.3	7.1	P < 0.01
Employed while in prison	79.3	60.0	72.0	P < 0.01
**Ever use drugs by drug type**				
Cannabis	89.1	98.8	92.7	P < 0.01
Amphetamines	66.0	95.0	76.9	P < 0.01
Cocaine	44.2	81.9	58.4	P < 0.01
Heroin	30.1	100.0	56.5	P < 0.01
Other opiates	18.9	66.9	36.9	P < 0.01
Tranquilisers	22.6	66.9	39.3	P < 0.01
Any drugs (other than heroin)	92.1	100.0	38.6	P < 0.01
Any drugs	92.5	100.0	95.3	P < 0.01
**Regularly (daily or almost daily) use drugs prior to prison by drug type**				
Cannabis	52.8	73.1	60.5	P < 0.01
Amphetamines	41.9	60.0	48.7	P < 0.01
Cocaine	23.8	56.9	36.2	P < 0.01
Other opiates	3.0	37.5	16.0	P < 0.01
Tranquilisers	6.4	35.6	17.4	P < 0.01
Any drugs (other than heroin)	69.1	92.5	77.9	P < 0.01
Any drugs	69.4	100.0	80.9	P < 0.01
**Offending history**				
Previously incarcerated	50.6	85.6	63.8	P < 0.01
Median number adult prison terms	2.0	4.0	2.0	P < 0.01
(+SD; Range)	(2.7; 1–20)	(2.8; 1–15)	(2.9; 1–20)	
Median years in prison at baseline	1.9	1.8	1.9	NS
(+SD; Range)	(4.2; 0.02-27.1)	(4.1; 0.03-23.2)	(4.2; 0.02-27.1)	
Median sentence length in years	3.9	3.1	3.6	NS
(+SD; Range)	(13.4; 0.5-113.9)	(15.4; 0.4-115.8)	(14.2; 0.4-115.8)	

*Abbreviations*: SD = standard deviation; NS = not significant.

Prisoners with a history of regular heroin use in the year before entering prison were significantly more likely to have ever used all other types of drugs, as well as regularly (daily or almost daily) using all other types of drugs prior to prison than prisoners who were not regular heroin users prior to entering prison. Similarly, prisoners who were regular heroin users prior to entering prison were significantly more likely to have been previously incarcerated and had twice as many adult prison terms than prisoners who were not regular heroin users.

### Smoking history, cessation behaviours and outcomes by regular heroin users

The mean ages for first smoking tobacco and for smoking tobacco on a daily basis were significantly lower for prisoners who were regular heroin users compared to those who were not regular heroin users (Table 2). There were no significant differences between participants who regularly used heroin and those who did not with regard to the quantity of tobacco smoked or nicotine dependence score. Participants who had regularly used heroin were significantly less likely to have attempted unsuccessfully to quit smoking in the past year, but there were no significant differences in the number of quit attempts.

**Table 2:** Smoking history, cessation behaviours and outcomes by regular use of heroin in year before prison

	**Not regularly used heroin % (N** = **265)**	**Regularly used heroin % (N** = **160)**	**Total men % (N** = **425)**	**P-value**
**Smoking behaviours**				
Mean age first smoked tobacco	14.1	13.0	13.7	P < 0.02
(+SD; Range)	(4.8; 5–39)	(3.1; 5–22)	(4.3; 5–39)	
Mean age first smoked tobacco daily	16.0	14.6	15.5	P < 0.01
(+SD; Range)	(4.6; 5–39)	(3.2; 7–30)	(4.2; 5–39)	
Mean years smoked tobacco	20.3	19.0	19.8	NS
(+SD; Range)	(11.2; 0–52)	(8.4; 3–46)	(10.2; 0–52)	
Mean carbon monoxide reading	14.1	14.5	14.3	NS
(+SD; Range)	(7.5; 1–43)	(8.2; 3–59)	(7.8; 1–59)	
Mean cigarettes smoked per day	23.1	23.3	23.2	NS
(+SD; Range)	(10.3; 3–75)	(8.9; 5–60)	(9.8; 3–75)	
Smoke 20+ cigarettes per day	67.6	74.4	70.1	NS
High tobacco dependence (Fagerström 6+)	80.8	86.3	82.8	NS
Share a cell with a smoker	32.2	35.6	33.5	NS
Smoke White Ox (loose tobacco)	97.0	97.5	97.2	NS
**Smoking cessation behaviours**				
Mean times tried to quit smoking	2.9	2.2	2.6	NS
(+SD; Range)	(8.6; 0–128)	(2.6; 0–20)	(7.9; 0–128)	
Quitting behaviours in past year				
Given up more than one month	14.0	11.3	12.9	NS
Tried to give up unsuccessfully	64.2	53.8	60.2	P < 0.04
Lower tar or nicotine content	7.9	4.4	6.6	NS
Reduced amount tobacco smoked	50.2	43.8	47.8	NS
Quit on purpose for 24 hours	57.0	48.1	53.7	NS
Any type of quitting behaviour	76.2	68.8	73.4	NS
**Smoking cessation intervention outcomes**				
Active NOR versus placebo NOR	49.4	46.9	48.5	NS
Quit smoking at 12 months: point prevalence	18.5	5.0	13.4	P < 0.01
Quit smoking at 12 months: continuous abstinence	16.6	3.8	11.2	P < 0.01
50% smoking reduction at 12 months	76.2	72.2	74.7	NS

*Abbreviations*: SD = standard deviation; NS = not significant; NOR = nortriptyline.

The outcomes of the smoking cessation intervention identified lower rates of quitting smoking for point prevalence (5.0% vs. 18.5%, P < 0.01) and continuous abstinence (3.8% vs.16.6%, P < 0.01) at 12-month follow-up for participants who were regular heroin users prior to prison. There were no significant differences between participants who were regular heroin users and those who were not with regard to having taken the study medication (NOR), nor with the outcome of reducing the quantity of tobacco smoked by 50% at 12 months.

### Predictors of smoking abstinence

Table 3 identifies potential predictors of smoking abstinence (both point prevalence and continuous abstinence) at 12 months, including socio-demographic, drug use and smoking behaviours which were significantly different between participants who, before entering prison, were regular heroin users and those who were not. The crude odds ratio for both models identified the following predictors of successfully quitting smoking at 12 months: being in prison for the first time, being in prison for five or more years, not being a regular heroin user and not being a regular user of other drugs (excluding heroin). When these predictors which were significant in the crude model were incorporated into the logistic regression model, not being a regular heroin user was the primary predictor for both point prevalence (OR = 3.81, 95% CI: 1.65-8.78) and continuous abstinence (OR = 4.29, 95% CI: 1.69-10.90).

**Table 3:** Predictors (crude and adjusted odds ratios) of smoking abstinence at 12 months

	**Point prevalence**		**Continuous abstinence**	
	**Crude**	**Adjusted**	**Crude**	**Adjusted**
	**Odds Ratio (95% CI)**	**Odds ratio (95% CI)**	**Odds Ratio (95% CI)**	**Odds ratio (95% CI)**
Active NOR versus placebo NOR	0.81 (0.46-1.42)	0.89 (0.49-1.62)	0.98 (0.54-1.77)	1.07 (0.57-2.00)
Age 35+ years	1.86 (1.06-3.25)†	1.46 (0.77-2.75)	1.64 (0.91-2.97)	1.36 (0.70-2.65)
Aboriginal origin	0.50 (0.19-1.31)	0.53 (0.19-1.48)	0.60 (0.23-1.56)	0.61 (0.22-1.72)
Left school no qualification	0.55 (0.31-1.01)	-	0.70 (0.38-1.29)	-
Institutionalised as a child	0.66 (0.36-1.21)	-	0.60 (0.32-1.16)	-
Homeless prior to prison	0.70 (0.21-2.39)	-	0.82 (0.24-2.82)	-
Employed while in prison	1.74 (0.87-3.48)	-	1.89 (0.89-4.02)	-
Tried to give up unsuccessfully	0.89 (0.51-1.57)	-	0.75 (0.41-1.36)	-
First time in prison	2.19 (1.25-3.84)†	1.47 (0.79-2.72)	2.52 (1.38-4.57)†	1.68 (0.88-3.20)
Incarcerated 5+ years at baseline	2.67 (1.44-4.96)†	2.30 (1.17-4.52)†	2.16 (1.11-4.20)†	1.88 (0.91-3.88)
Not regular drug use (excluding heroin) in year prior to prison	2.37 (1.33-4.19)†	1.31 (0.65-2.62)	2.39 (1.30-4.39)†	1.33 (0.64-2.75)
Not regular heroin use in year prior to prison	4.31 (1.98-9.36)†	3.81 (1.65-8.78)†	5.11 (2.13-12.29)†	4.29 (1.69-10.90)†

*Abbreviations*: NOR = nortriptyline † P < 0.05.

## Discussion

This paper concludes that heroin use in the year before entering prison is associated with a reduced likelihood of stopping smoking at 12 months following a multi-component smoking cessation intervention. This finding remained significant after controlling for regular use of drugs other than heroin, which strengthens the specificity of this association. Though there are a number of studies identifying the importance of implementing smoking cessation interventions among people in opioid treatment programs [5,26,27], no studies were found which investigated regular heroin use as a possible risk factor for an unsuccessful quit smoking attempt. As nearly half of smokers die of a tobacco-related illness [28] and the annual mortality rate for heroin-dependent people is a further 2% [29], the combined risk factors of smoking and heroin dependence have important public health implications.

The findings of this study suggest that future smoking cessation interventions should consider identifying concurrent drug addictions (particularly heroin) and identify strategies to deal with all drug problems at the same time. There is a growing body of evidence supporting multiple behaviour change interventions which can be applied to addressing smoking cessation and drug use [30,31]. A recent meta-analysis of the outcomes of smoking cessation interventions among participants in drug and alcohol treatment identified that smoking cessation interventions were associated with a 25% increase in long-term drug and alcohol abstinence [32], a finding supported by other studies [33,34]. As many persons undergoing treatment for heroin dependence in the community may later enter prison [35], strong support to quit smoking should be provided as a routine in all drug treatments for heroin dependence.

Prisoners with a history of regular heroin use in the year prior to entering prison had significantly poorer social determinants of health such as poor educational attainment, higher involvement in out-of-home care, and were also more likely to be homeless. These determinants are also important risk factors for smoking tobacco, and they reduce the success rate for smoking cessation interventions [36,37]. Further, participants who used heroin regularly prior to prison also were significantly more likely to have been previously incarcerated and had a median of twice as many previous times in prison compared to those who had not used heroin regularly. Increased involvement in the criminal justice system is likely to exacerbate poverty, homelessness and other social disadvantages which are associated with tobacco smoking [38].

Another key characteristic of prisoners who had regularly used heroin in the year before entering prison is that they started smoking at a younger age and reported significantly more other drug use than prisoners who did not report regular heroin use. It is well documented that drug and alcohol treatment services often fail to address underlying tobacco dependence [39-41]. Some reasons for this include inadequate staff knowledge and training for providing smoking cessation interventions, high smoking prevalence rates among staff, and ambivalent attitudes and beliefs related to tobacco among staff [41]. These barriers to providing smoking cessation among people with drug and alcohol problems may be addressed through providing enhanced staff education and training in smoking cessation, supporting staff to quit smoking and creating a smoke-free environment for drug and alcohol treatment services [42]. The magnitude of the barriers to treatment provision and uptake for prisoners coupled with the major adverse biological and social factors associated with severe drug dependence [43], suggests that very powerful interventions to assist smoking cessation will be required to increase quit rates in this population.

### Limitations

One limitation of this study is that heroin use was assessed by participant self-report, so may not reflect the true proportion of prisoners who had ever or recently used heroin. Some prisoners may have been concerned about admitting to an illegal behaviour while in prison. However, the use of independent research nurses was expected to improve the confidence of participants that their responses would be kept confidential. This study was also limited by only including males so cannot necessarily be generalised for female prisoners. Also, this study was also limited by its stringent inclusion and exclusion criteria, which were necessary in ensuring safe administration of nortriptyline, but which excluded a high proportion of men interested in quitting smoking. It is not possible to determine if the men who were eligible were more or less likely to use heroin than those who were not so the generalisability of these findings must be considered with caution. Another limitation of the study is that participants were not asked if they were on an opioid substitution program or if they were currently using heroin so we cannot determine if this had an impact on smoking cessation success. Despite these limitations, this study has a number of strengths including: a strong follow-up rate of 80% at 12 months; recruitment across nearly all male prisons in NSW; adherence to a rigorous study protocol; and utilising a tailored multi-component smoking cessation intervention that was piloted among prisoners [3].

## Conclusions

The key finding of this study was that, before entering prison, prisoners who had regularly used heroin were significantly less likely to have successfully quit smoking than prisoners who did not report regular heroin use. This suggests that smoking cessation interventions for prisoners should take into account prisoners’ recent drug (particularly heroin) use in order to develop an effective intervention which tackles both smoking and illicit drug use. More research may assist in determining if these interventions should occur as a combined model or separately and the optimal timing of their implementation.

Many prisoners wish to quit smoking. Incarceration presents an opportune time to attempt engaging prisoners in smoking cessation interventions. More research is needed to determine how best to support prisoners who regularly used heroin to quit smoking. Some evidence suggests that when people are able to quit smoking, they are better able to stop using illicit drugs, which may result in reductions in re-offending, as well as significant improvements in health and well-being.

## Abbreviations

SD: Standard deviation; NS: Not significant; NOR: Nortriptyline; NSW: New South Wales.

## Competing interests

The authors declare that they have no competing interests.

## Authors’ contributions

RR, KW, TB and AW were involved in all aspects of this study from conceptualisation, funding, and development of questionnaire for baseline and follow-up assessments. DI and VA joined the study and made substantial contributions to data acquisition. DI conducted the data analysis and prepared the draft manuscript. All authors have been involved in data interpretation, finalising the manuscript, and have given final approval of the version to be published.

## Pre-publication history

The pre-publication history for this paper can be accessed here:

http://www.biomedcentral.com/1471-2458/13/1200/prepub
